# An extragastrointestinal stromal tumor originating from the seminal vesicles: A case report and review of the literature

**DOI:** 10.3892/ol.2013.1496

**Published:** 2013-07-26

**Authors:** YI HOU, YINHUAI WANG, RAN XU, DUO LI, XIAOKUN ZHAO

**Affiliations:** 1 Department of Urology, The Second Xiangya Hospital, Central South University, Changsha, Hunan 410011, P.R. China; 2 Department of Pathology, The Second Xiangya Hospital, Central South University, Changsha, Hunan 410011, P.R. China

**Keywords:** extragastrointestinal stromal tumor, pathology, seminal vesicles

## Abstract

The present study reports a case of an extragastrointestinal stromal tumor (EGIST) originating from the seminal vesicles. A 74-year-old male patient with a tumor in the seminal vesicles underwent a radical spermatocystectomy due to an increased defecation frequency and a huge mass in the seminal vesicles. Ultrasonography and computed tomography (CT) initially diagnosed the mass as a tumor originating from the prostate. However, the mass was ultimately confirmed as an EGIST from the seminal vesicles following a laparotomy. According to the size, mitotic activity, cellularity, necrotic situation and immunohistochemical data, the tumor belonged to a low-risk group. No recurrence or metastasis has been identified during six years of follow-up observations. To the best of our knowledge, this is the first study to report this particular pathological type of EGIST.

## Introduction

Gastrointestinal stromal tumors (GISTs) are one of the most common types of mesenchymal tumor of the gastrointestinal tract (1–3% of all gastrointestinal malignancies). GISTs were first reported by Mazur and Clark in 1983 (1) and were typically defined as tumors whose behavior is driven by mutations in the Kit or PDGFRA genes. Barium fluoroscopic examinations and computed tomography (CT) scans are often undertaken to determine a diagnosis of GIST. Immunohistochemistry (specific antibodies that stain the CD117 molecule) is also adopted to confirm the diagnosis when a GIST is suspected.

Extragastrointestinal stromal tumors (EGISTs) are neoplasms with overlapping immunohistological features, occurring in the abdomen outside the gastrointestinal tract with no connection to the gastric or intestinal wall (2). Similar to GISTs, these tumors consistently exhibit positive staining for CD117 (2). High cellularity, mitotic activity and necrosis have been shown to be associated with a statistically significant increased risk for an adverse outcome (4). Normally, EGISTs originate from particular sites, including the pancreas, greater omentum, retroperitoneum, mesocolon, mesentery of the small intestine or pelvis (3–6), but they may also exist in the urological system. For instance, certain cases of primary EGIST have been reported to originate from the bladder, urethra and prostate (7–10). The present study reports the results of clinical and microscopic examinations, including immunohistochemical studies, of an EGIST from the seminal vesicles. To the best of our knowledge, the present case is the first to report this pathological type of EGIST. Written informed consent was obtained from the patient.

## Case report

**Figure 1:**
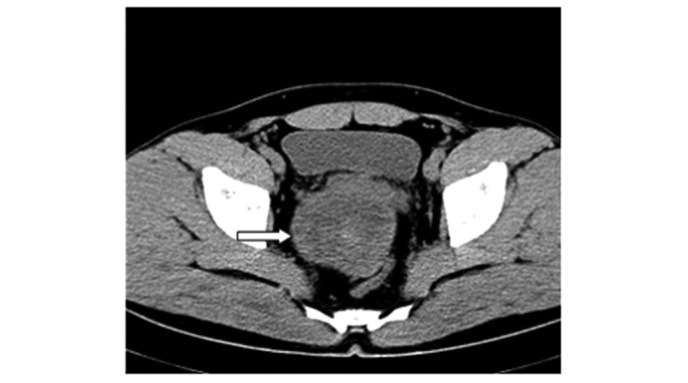
CT image demonstrating a 76×77×74-mm cystic mass and ring-like subcapsular high density district (arrows), without a connection with the adjacent tissues. CT, computed tomography.

A 74-year-old male was admitted to the Second Xiangya Hospital (Central South University, Changsha, Hunan, China) in October 2005 due to an increased defecation frequency for a duration of more than one year. For one year prior to being admitted, the patient had observed a defecation frequency that had increased from once per day to six-seven times per day without precipitating causes and accompanied by stool reduction. The stool was yellow, but retained its shaped. The patient had no history of a frequent/urgent requirement to urinate, gross hematuria or abdominal pain. The prostate could not be palpated clearly when performing the physical examination, though a huge mass was identified behind the prostate during the rectal examination. The mass was medium-hard without unusual palpable nodules and tenderness. The other physical examinations were normal. Ultrasonography revealed that the boundary of the prostate was unclear and that it was 39×25×23 mm in size. A thick-walled inhomogenous hypoechoic cystic mass (76×77×74 mm), in which the thickness of the cyst wall was uneven, was detected posterior to the prostate. The level of blood prostate specific antigen (PSA) was normal, while the pelvic CT (Fig. 1) revealed that the diameters of the mass were 77 mm from top to bottom and 76 mm from right to left. A ring-like subcapsular high-density district was observed in the mass and there was a mixed high and low density and punctate calcification. The mass was not connected to the adjacent tissues and appeared to originate from the prostate. The contrast enhanced CT also demonstrated non-uniform changes. The bladder was filling well and the wall was normal. No swollen lymph nodes were identified in the cavity.

**Figure 2:**
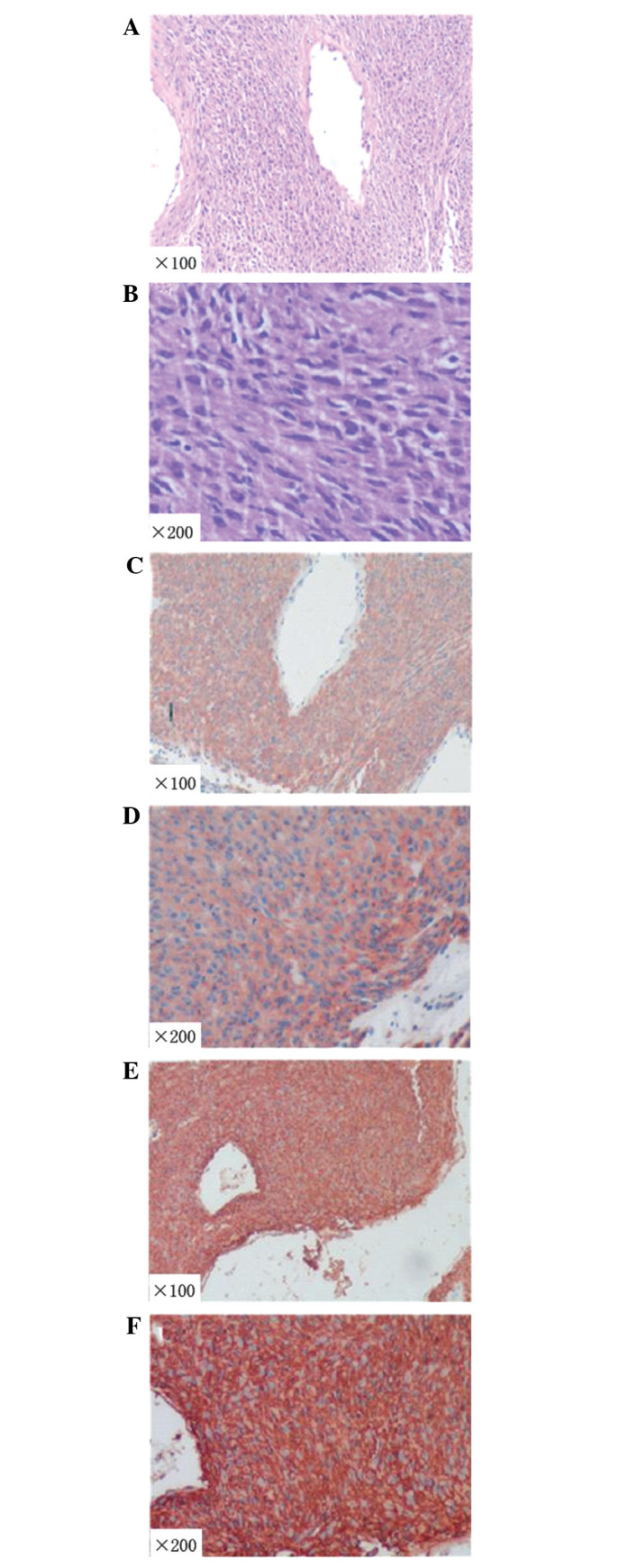
Histological and immunohistochemical features of the tumor. Light microscopy showing that the mass was composed of abundant spindle tumor cells which were arrayed in bundles around the blood vessels. Mitosis was rare in these cells: (A) ×100 magnification; and (B) ×200 magnification (HE staining). Immunohistochemical staining indentified that the tumor cells displayed strong positive expression of CD117: (C) ×100 magnification; and (D) ×200 magnification (AEC staining). The tumor cells also displayed strong positive expression of CD34: (E) ×100 magnification; and (F) ×200 magnification (AEC staining).

A laparotomy through an abdomen incision revealed a large, slightly capsulated mass that arose from the seminal vesicle but not the prostate, without connecting to the gastrointestinal tract. The size of the mass was 8×7×7 cm and the capsule boundary was clear, without adherence to the organs around the seminal vesicles. The mass was removed using the no-touch technique. A biopsy and immunophenotype staining were performed. The texture of the mass was moderate and the surface was smooth with a fibrous membrane. Light microscopy examination revealed hemorrhage and necrosis with rich and crowded cells. Karyokinesis was rare and the mitotic index was <10 mitoses per 50 high power fields (HPF). The histopathological diagnosis was of a mesenchymal tumor originating from the seminal vesicle. The immunohistochemical studies revealed that the cells were strongly positive for CD34, CD117, PDGFRA and Vim (Fig. 2) and negative for HHF35, smooth muscle actin (SMA), S-100 protein and creatine kinase (CK). These results strongly supported a diagnosis of a low-risk EGIST of the seminal vesicle. A point mutation analysis for a KIT protein mutation was not performed due to its unavailability at the Departments of Urology and Pathology, The Second Xiangya Hospital. The patient received 600 mg imatinib orally, taken once daily with food for 6 months. Currently, the patient is being followed-up and has not experienced a recurrence or metastasis for 6 years.

## Discussion

GISTs are a type of mesenchymoma and are always located in the GI tract. Generally, the tumors should be classified as EGISTs or GISTs by whether they originate from the GI tract or grow out of the serous membrane. GISTs are characterized by the predominant expression of the c-kit protein and CD34. The combination of the immunohistochemical examinations for c-kit and CD34 are the most reliable markers for the pathological diagnosis of GIST thus far (11).

EGISTs arise from outside the GI tract, but have similar histological features to their GI counterparts, including the morphology, immunophenotype and molecular genetic characteristics. However, the site, growth methods, imaging characteristics and prognosis of EGISTs vary from those of GISTs. In general, EGISTs are an aggressive group of tumors sharing a similarity to GISTs arising in the distal GI tract. EGISTs are rare tumors. From the published literature, it may be observed that the most common EGISTs originate from the omentum, mesentery, retroperitoneum (2,12–15) and lymph nodes (16). The probability of EGISTs occurring in the urological system is very low and to date, there have not been any studies with regard to EGISTs from the seminal vesicles. In the present case, no tumors were identified inside or outside the intestinal tract. Primarily, the CT and ultrasonography indicated that the tumor may have originated from the prostate, but subsequently, the laparotomy revealed that the tumor was from the seminal vesicle. The strongly positive staining of the tumor cells for CD34 and CD117 also suggested that it may be classified as an EGIST. The clinical manifestations of EGISTs from the prostate and seminal vesicle may be similar due to the close anatomical site, therefore, a misdiagnosis was made prior to the laparotomy.

Predicting the malignant potential of tumors is significant for their treatment. Mitotic activity, cellularity and the presence of necrosis have been shown to be associated with a worse outcome (4). A high mitotic rate (>5/50 HPF) and Ki-67 labeling index (>10%) may lead to a significantly poorer outcome. Reith *et al* observed that a mitotic rate of >2/50 HPF, the presence of necrosis and a high cellularity was useful in predicting the biological behavior of EGISTs (4). However, a study by Yamamoto *et al* suggested that C-kit gene mutations were not correlated with the prognosis in patients with EGISTs (17). CD117 is a transmembrane receptor and a growth factor expressed in hematopoietic stem cells, mast cells, melanocytes and a neural-related population of GI mesenchymal cells, the Cajal cells, that are located in the muscular layers (12). The PDGFRA gene also plays a role in a small population of EGISTs. Kim *et al* reported the first case of a tumor specimen that contained multiple mutations of c-kit and PDGFRA (18). In the present case, the large size, presence of necrosis and positive expression of CD117, CD34 and PDGFRA in the tumor cells suggested a malignant potential, while the low mitotic rate (<10/50 HPF) did not favor this conclusion. In addition, no tumor metastasis was observed during the laparotomy. Thus, this tumor was classed as low-risk. No recurrence or metastases have been identified during six years of follow-up observations.

An EGIST of the seminal vesicles must be differentiated from a specific interstitial tumor of the seminal vesicles, including unconfirmed interstitial hyperplasia. The differentiation may be achieved according to the association between the hyperplastic interstitial composition and the body of the seminal vesicles. Immunohistochemical results are the most effective strategy for differentiating between the two types of tumor (9). Normally, CD34 and C-kit gene staining are strongly positive in an EGIST originating from the seminal vesicles, while only CD34 is positive in the hyperplasia of the seminal vesicles. The present case was misdiagnosed as a prostate tumor prior to a laparotomy since the huge tumor was adjacent to the prostate and the CT scan was not able to distinguish the boundary. Thus, the immunohistochemical method of diagnosis of EGIST should be performed prior to laparotomy.

The present case of a stromal tumor in the seminal vesicles may be defined as a GIST-type malignant stromal tumor, rather than a specific interstitial tumor or hyperplasia of the seminal vesicles. This diagnosis was made by the fact that the histological and immunological phenotypes were similar to those of a GIST. The present study may provide clinicians with a reference point for the differential diagnosis of tumors of the prostate and the seminal vesicles.

## References

[1] Mazur MT, Clark HB (1983). Gastric stromal tumors. Reappraisal of histogenesis. Am J Surg Pathol.

[2] Franzini C, Alessandri L, Piscioli I (2008). Extra-gastrointestinal stromal tumor of the greater omentum: report of a case and review of the literature. World J Surg Oncol.

[3] Barros A, Linhares E, Valadão M (2011). Extragastrointestinal stromal tumors (EGIST): a series of case reports. Hepatogastroenterology.

[4] Reith JD, Goldblum JR, Lyles RH, Weiss SW (2000). Extragastrointestinal (soft tissue) stromal tumors: an analysis of 48 cases with emphasis on histologic predictors of outcome. Mod Pathol.

[5] Liao JM, Mayer WA, Kim MM, Link RE (2011). Robot-assisted laparoscopic excision of a pelvic extragastrointestinal stromal tumor: a case report and literature review. Can J Urol.

[6] Zhang W, Peng Z, Xu L (2009). Extragastrointestinal stromal tumor arising in the rectovaginal septum: report of an unusual case with literature review. Gynecol Oncol.

[7] Krokowski M, Jocham D, Choi H (2003). Malignant extragastrointestinal stromal tumor of bladder. J Urol.

[8] Mekni A, Chelly I, Azzouz H (2008). Extragastrointestinal stromal tumor of the urinary wall bladder: case report and review of the literature. Pathologica.

[9] Yinghao S, Bo Y, Xiaofeng G (2007). Extragastrointestinal stromal tumor possibly originating from the prostate. Int J Urol.

[10] Shin HS, Cho CH, Kum YS (2011). Extragastrointestinal stromal tumor of the urinary bladder: a case report. Urol J.

[11] Vij M, Agrawal V, Pandey R (2011). Malignant extra-gastrointestinal stromal tumor of the pancreas. A case report and review of literature. JOP.

[12] Liu H, Li W, Zhu S (2009). Clinical images. Extragastrointestinal stromal tumor of lesser omentum mimicking a liver tumor. Am J Surg.

[13] Terada T (2008). Primary multiple extragastrointestinal stromal tumors of the omentum with different mutations of c-kit gene. World J Gastroenterol.

[14] Llenas-García J, Guerra-Vales JM, Moreno A (2008). Primary extragastrointestinal stromal tumors in the omentum and mesentery: a clinicopathological and immunohistochemical study. Hepatogastroenterology.

[15] Park SS, Min BW, Kim WB (2005). Malignant extragastrointestinal stromal tumor of retroperitoneum. Acta Oncol.

[16] Kim HJ, Park C, Cho SY (1997). A case of extragastrointestinal anisakiasis involving a mesocolic lymph node. Korean J Parasitol.

[17] Yamamoto H, Oda Y, Kawaguchi K (2004). c-kit and PDGFRA mutations in extragastrointestinal stromal tumor (gastrointestinal stromal tumor of the soft tissue). Am J Surg Pathol.

[18] Kim JH, Boo YJ, Jung CW (2007). Multiple malignant extragastrointestinal stromal tumors of the greater omentum and results of immunohistochemistry and mutation analysis: a case report. World J Gastroenterol.

